# Gender differences in vitiligo: psychological symptoms and quality of life assessment description

**DOI:** 10.3389/fpsyg.2023.1234734

**Published:** 2023-12-20

**Authors:** Tonia Samela, Walter Malorni, Paola Matarrese, Gianfranco Mattia, Stefania Alfani, Damiano Abeni

**Affiliations:** ^1^Clinical Psychology Unit, Istituto Dermopatico Dell’Immacolata, (IDI) IRCCS, Rome, Italy; ^2^Clinical Epidemiology Unit, Istituto Dermopatico Dell’Immacolata, (IDI) IRCCS, Rome, Italy; ^3^Center for Global Health, Università Cattolica del Sacro Cuore, Rome, Italy; ^4^Center for Gender-Specific Medicine, Istituto Superiore di Sanità (ISS), Rome, Italy

**Keywords:** gender differences, vitiligo, skin disease, psycho-dermatology, psychology assessment, quality of life assessment, literature review

## Abstract

**Objective:**

Assuming that the difference exist in the manifestation of psychological suffering among genders, the purpose of this review is to summarize the current knowledge on gender differences in vitiligo quality of life and psychological assessment.

**Methods:**

We searched in PubMed, Scopus, and Web of Science databases for original articles in English language. Results were screened according to the Preferred Reporting Items for Systematic Reviews and Meta-Analyses (PRISMA checklist).

**Results:**

The study yielded 107 results; 12 articles have been evaluated as eligible. Each eligible study has been screened and analyzed. The study’s qualitative evaluation revealed that 8 papers were classifiable as satisfactory, 4 were classifiable as unsatisfactory. The agreement between the coders was high (% agreement = 84.6%; Cohen’s kappa = 0.79). All considered researches (100%) were cross-sectional studies, based on self-report questionnaires. From our analysis, women with vitiligo had a higher risk to experience lower quality of life, and worse mental health in a wide range of psychopathology symptoms than men. A wide heterogeneity of tools is used to investigate the quality of life and psychological symptoms among these patients.

**Conclusion:**

Unfortunately, there are few explanatory models proposed in the literature to rationalize these findings. It will be important to investigate in further researches the specific influence of known risk factors for psychopathology in this population to better explore these phenomena.

## Introduction

Vitiligo is a complex immune-mediated skin disorder characterized by the progressive destruction of epidermal melanocytes, that result in well-defined depigmented patches (Alikhan et al., 2011). Vitiligo may occur everywhere on the body (Spritz and Santorico, 2021). The prevalence of the disease differs worldwide (Taïeb et al., 2007; Sampogna et al., 2008; Krüger and Schallreuter, 2012; Sheikh et al., 2022).

Vitiligo is one of the psycho-dermatological disorders that causes no direct physical impairment or pain, but it has a significant impact on the personal and social life due to cosmetic disfigurement (Sampogna et al., 2008). Vitiligo is a chronic skin disease (Speeckaert and van Geel, 2017; Shourick et al., 2021; Eleftheriadou et al., 2022). Quantitative and qualitative studies assessed that people who suffered from vitiligo are exposed to a higher risk of live through relevant issues like shame, discomfort, embarrassment, and body uneasiness (Tomas-Aragones and Marron, 2016), and may try to hide lesions, especially when they appear on the exposed part of the body (Picardi et al., 2001b; Lai et al., 2017; Grimes and Miller, 2018). The presence of cognitive biases of stigma (Bonotis et al., 2016; Heng, 2022), the worry about how other people will react to the manifestation of the disease, and the poor body-image could result in social anxiety (Salman et al., 2016) or self-isolation behaviors (Grimes and Miller, 2018) that could trigger or worsen the depressive disorder (Jafferany and Pastolero, 2018). Several studies stressed the evidence that chronic skin diseases can lead to depression, which in turn increases the risk of suicide ideation, attempted suicide, or suicide (Picardi et al., 2006; August and Sorkin, 2010; McDonald et al., 2018; Padmakar et al., 2022).

Depressive or mood disorders frequently do not occur alone, are in fact commonly associated with anxiety symptomatology (Lai et al., 2017). These anxiety symptoms are characterized by feelings of worry and uneasiness, that lead to frequent episodes of overreaction to problems that -according to the patient’s perception- appear to be unsurmountable (Hofmann, 2005). As a result, these cognitive processes, merged with the assumption of non-recoverability of vitiligo, could trigger hopeless thoughts, that in turn increase the risk of depression (Ribeiro et al., 2018).

Taking into account the substantial load of psychological impairment reported by these patients, and assuming -based on the published research- that relevant gender differences exist in the manifestation of psychological suffering (Rosenfield and Mouzon, 2013; Juvrud and Rennels, 2017; Darnon et al., 2018), the purpose of this review is to depict the actual knowledge on gender differences in vitiligo, from the available data in published literature up to November 2022, with a focus on psychosocial aspects of vitiligo.

## Materials and methods

### Literature search

This review was prepared according to the Preferred Reporting Items for Systematic Reviews and Meta-Analyses (PRISMA) statement. We systematically searched peer-reviewed original articles published in English in the following databases: PubMed, Scopus, and Web of Science, without time restriction. The last search was performed on November 21, 2022. To perform the literature analysis, we combined the terms: “gender,” “vitiligo,” “psychology,” using the Boolean connector “and” (i.e., ((gender) AND (vitiligo)) AND (psychology)). Secondly, we assessed the abstracts of all potentially pertinent articles to check if they met the eligibility criteria. The study quality assessment was carried out independently by two reviewers (TS and DA).

### Eligibility criteria

In this review we included all the cross-sectional, case–control, or cohort studies that assessed gender differences in patients with vitiligo using validated methods. Case reports, case series, review articles, letters to the editor, and psychometric studies were excluded. The databases selected to perform this work did not report gray literature; for this reason, unpublished studies were not included in this review. The data found in conference papers or abstract books have not been reported as well, because are usually reported later in published articles.

### Selection of studies and data extraction

To select potential studies, the title, the abstract and keywords of each article found in each database were reviewed. In this phase article duplicates (*N* = 77) have been removed. Eighteen records were further excluded because the full-text was not available, or results were not focused on gender differences, or the full text was not in English. Twelve studies were included in the qualitative synthesis, and all of them were included in the quantitative analysis.

Data from the studies of interest were extracted and inserted in a structured dataset; core information about each publication have been addressed using a standardized reporting form. All the authors reviewed the summary table of the included studies.

### Quality assessment

The quality of the eligible articles was independently assessed by the first and the last authors (TS and DA) using the Newcastle-Ottawa Scale (NOS) modified for the assessment of the quality of cross-sectional studies. This methodological instrument has been previously adopted in meta-analytic epidemiology studies (Stang, 2010) and in systematic reviews on immune-mediated chronic skin diseases (Machado et al., 2019). The scores are comprised between 0 and 10, and a higher score is indicative of a higher quality of the study (i.e., Very Good Studies: 9–10 points; Good Studies: 7–8 points; Satisfactory Studies: 5–6 points; Unsatisfactory Studies: 0–4 points). In case of incongruity between reviewers, agreement was reached after in-person discussion.

## Results

**Figure 1:**
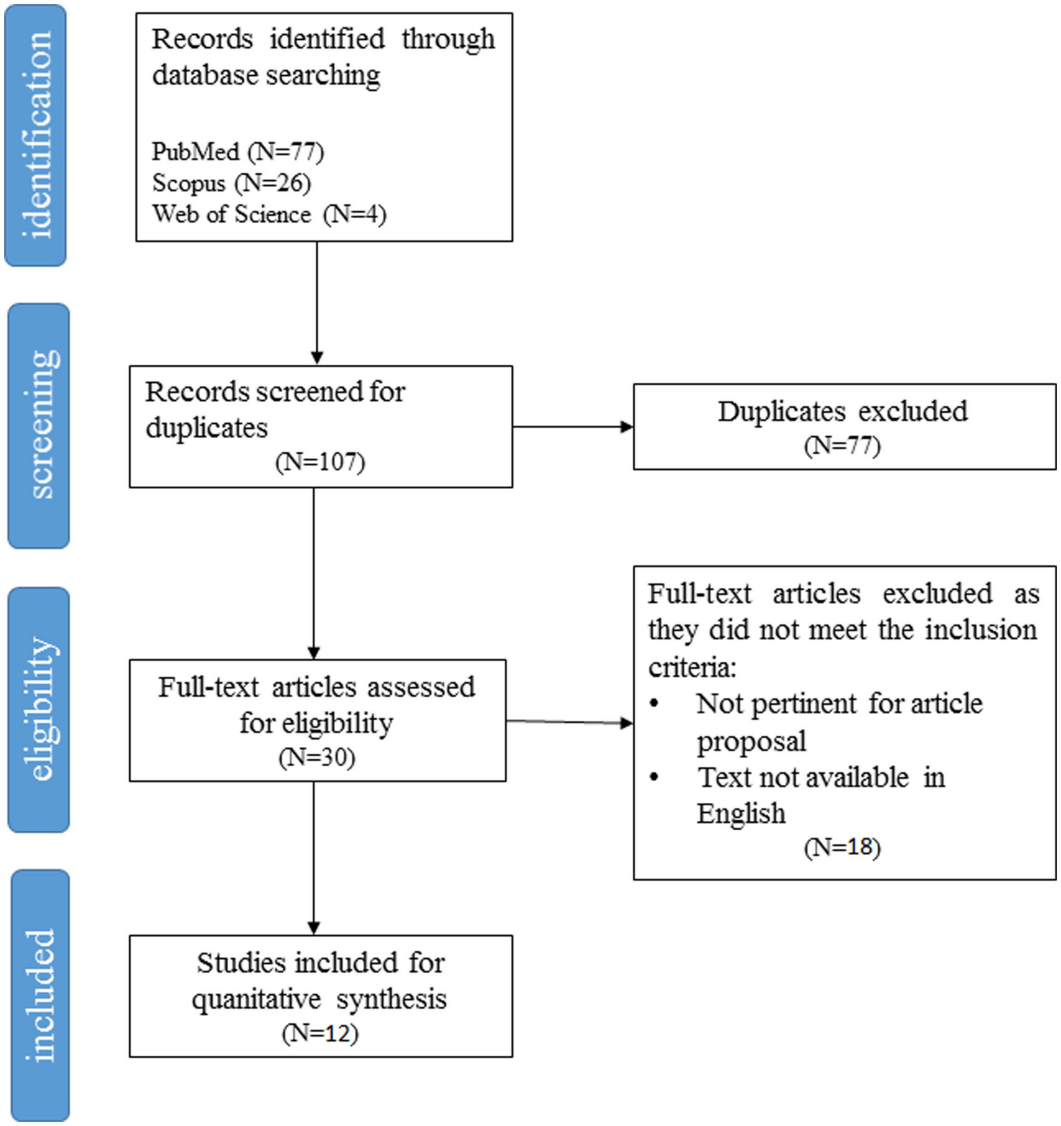
PRISMA flow diagram of psychosocial studies on gender differences in vitiligo retrieved using the search string detailed in the Methods section.

The flowchart shown in Figure 1 details each step of the study selection process. The results in each step have also been specified in the figure. Quantitative synthesis of the selected study features has been reported in Table 1. Inter-coder agreement was high when the independent coders (T.S. and D.A.) examined the full text of the 12 articles identified as eligible, the agreement between the coders was high (% agreement = 84.6%; Cohen’s kappa = 0.79). Disagreements were resolved through discussion. According to McHugh (2012), from the study’s qualitative evaluation (McHugh, 2012), eight papers are classifiable as satisfactory studies, while five are classifiable as unsatisfactory. A specifically description of each study’s evaluation is reported in Table 2.

**Table 1:** Summary of collected and analyzed data from psychosocial studies on gender differences in vitiligo included in this review.

First author, country, and year	Setting and sample size	Purpose	Study design	Criteria of eligible population	Assessment tools	Gender differences	Psychologic syndrome/symptomatology
Abdullahi, U., Nigeria, 2021	Dermatology Clinic of Ahmadu Bello University Teaching Hospital, Nigeria Overall *N* = 77 Male *N* = 32 Female *N* = 45	To assess the QOL among Nigerians with vitiligo using VitiQoL. To determine those clinical and sociodemographic factors that affect the QOL in the study participants	cross-sectional study	Patients aged from 18 to 67 years old who attended the Dermatology Clinic of Ahmadu Bello University Teaching Hospital Nigeria, between July 2019 and March 2020	VASI*^1^ VitiQoL*^2^	Mean VitiQoL score was 30.51 ± 15.74 (with a range of 3–64). The QOL is significantly more impaired in females than in males (*p* = 0.037). Stigma component of VitiQoL was the major contributor to the high mean VitiQoL (*p* < 0.001)	Health-related quality of life impairment
Al Robaee A. A., Saudi Arabia, 2007	Qassim Medical College clinics Overall *N* = 109 Male *N* = 61 Female *N* = 48	To determine the quality of life in Saudi patients with vitiligo. To detect the variables that could influence it by using the Dermatology Life Quality Index (DLQI)	Cross-sectional study	Patients recruited from Qassim Medical College clinics between November 2004 and September 2006. Age ranged from 18 to 47 years old.	Arabic adaptation of DLQI*^3^	No statistical difference between males and female in DLQI scores. Significative differences among men and women was found between light therapy and topical treatment patients; women were more impaired in personal relationships, sexual activities, social life and in choosing clothes (*p* < 0.05).	Health-related quality of life impairment
Dabas., G., India, 2020	The pigmentary clinic of postgraduate institute of medical education and research, Chandigarh Overall *N* = 272 Vitiligo *N* = 95 Male *N* = 34 Female *N* = 61 Acquired Dermatological Hyperpigmentation *N* = 91 Male *N* = 21 Female *N* = 70 Melasma *N* = 86 Male *N* = 28 Female *N* = 58	To assess the burden of all major psychiatric disorders: depression, anxiety disorder, somatization disorder, eating disorder and alcohol abuse among patients with pigmentary abnormalities	Cross-sectional study	Age ≥ 18 years, presence of facial lesions or lesions on exposed body parts (face, arms, and dorsum of hands), ability to read and understand the self-assessment questionnaires.	MASI*^4^ VASI*^1^ DPASI plus*^5^ Hindi and Punjabi language DLQI*^3^ PRIME-MD PHQ*^6^ PHQ-9*^7^ PHQ-15*^8^ GAD-7*^9^	Males and females scores did not differ significatively (DLQI *p* = 0.13; PHQ-9 *p* = 0.11; PHQ-15 *p* = 0.96; GAD-7 *p* = 0.54)	Alcohol abuse, Anxiety disorder, Depression, Eating disorders, Somatization disorder
Do Bú, E., Brazil, 2002	Patient enrolled by online platform Overall *N* = 325 Male *N* = 58 Female *N* = 267	To verify how both stress and rumination would affect the relationship between neuroticism, anxiety, and depression symptoms. To test if the participants’ gender could function as a moderator in these relationships, proposing moderated mediation hypotheses	Cross-sectional study	To be older than 18 years; to be Brazilian citizens; to be diagnosed with Vitiligo by a dermatologist.	RRS-VR*^10^ Big Five Personality Factors Inventory DASS*^11^	Differences between genders concerning neuroticism [*F*(1, 323) = 7.64; *p* = 0.006, *d* = 0.40]; stress [*F*(1, 323) = 10.28; *p* = 0.001, *d* = 0.48]; rumination (brooding) [*F*(1, 323) = 7.53; p = 0.006; *d* = 0.41]; anxiety [*F*(1, 323) = 10.18; *p* = 0.002; *d* = 0.43]; depression [*F*(1, 323) = 12.72; *p* = 0.001, *d* = 0.55]; women showed a significant higher averages for all of this constructs. Hierarchical multiple regression tested, evinced that participants’ female gender and neuroticism predict anxiety and depression. Analyses of moderated mediation showed significant interactions between gender and stress (*b* = 0.30; SE = 0.14; *p* = 0.04), and between gender and brooding (*b* = −0.29; SE = 0.09; *p* = 0.001); in such a way that the mediation by stress was stronger for women (indirect effect: *b* = 0.49; SE = 0.04; CI 95% = 0.40; 0.57) than for men (indirect effect: *b* = 0.28; SE = 0.07; CI 95% = 0.15; 0.45), and the brooding mediation was stronger for men (indirect effect: *b* = 0.23; SE = 0.07; CI 95% = 0.10; 0.38) than for women (indirect effect: *b* = 0.07; SE = 0.02; CI 95% = 0.03 0.12). Therefore, the individuals’ gender seemed to be an important variable in the processes that link neuroticism to anxiety and depression through stress and rumination’s dimensions.	Anxiety, Brooding, Depression, Neuroticism, Rumination, Stress
Ingordo, V., Italy, 2014	Nine Dermatological Hospital Centers (Cremona, Bologna, Reggio Emilia, Siena, Grosseto, Caserta, Taranto, Gagliano del Capo, Catania) Overall *N* = 161 Male and female percentage not specified	To estimate the Quality of Life in a fairly large sample of Italian vitiligo patients by using the Dermatology Life Quality Index (DLQI)	Multicenter observational study	–	The Italian version of the DLQI*^3^	Gender (female) was significantly associated with DLQI >5.	Health-related quality of life impairment
Kent, G., United Kingdom, 1996	Patient enrolled by mail in the context of Vitiligo Society, UK Overall *N* = 614 Men *N* = 150 Female *N* = 464	To assess its applicability in a non-clinic sample of vitiligo sufferers and to measure its relationship with questionnaires designed to measure a variety of psychological and demographic factors	Cross-sectional study	From an age ≥ 16 years old, to 81 years old	Ginsburg and Link’s psoriasis stigma questionnaire; Rosemberg’s scale; GHQ-12*^12^ DLQI*^3^	Males and females scores did not differ significatively (means = 4.65 and SD ±4.88, *t*(612) = 0–51, *p* > 0.50)	Health-related quality of life impairment
Krüger, C., Germany, 2015	Institute for Pigmentary Disorders (IFPD), Greifswald Vitiligo Overall *N* = 96 Men *N* = 35 Female *N* = 61 Healthy Controls Overall = 23 Men = 13 Female = 10	To explore Quality of Life, coping and depression in adults with vitiligo. To compare the results with those of healthy adult.	Cross-sectional study	Consecutive patients who booked for an appointment at the IFPD from beginning of September to end of December 2012	DLQI*^3^ ACS*^13^ BDI*^14^ additional questions to explore Quality Of Life	In all skin disease-specific scales, there were no significant differences between men and women. Female patients were slightly more anxious-depressed (ACS anxious depressive mode *p* = 0.026; BDI *p* = 0.048)	Anxious/depressive mood, Depression, Helplessness, Quality of Life Social anxiety/avoidance, Coping.
Ning, X., China, 2022	Second Affiliated Hospital of Xi’an Jiaotong University Overall *N* = 117 Male *N* = 49 Female *N* = 68	To investigate the psychological and behavioral status in patients with vitiligo in China through psychological and behavioral related questionnaires, analyze the influencing factors of psychological and behavioral problems, and provide theoretical basis for comprehensive treatment and clinical intervention of vitiligo.	Cross-sectional study	Inclusion criteria were: 1. All patients were over 18 years old. 2. The diagnosis of vitiligo was made by two dermatologists and wood lamp. 3. All patients had no previous history of mental illness. 4. All patients had no cognitive orientation disorder, had primary school education or above, and could understand the meaning of the questionnaires. 5. All patients voluntarily participated, and expressed their willingness to cooperate to complete the questionnaires carefully. Exclusion criteria: 1. Patients were under 18 years old. 2. Patients with other skin diseases or major physical or neurological diseases (cancers, heart problems, liver and kidney dysfunctions, epilepsy, etc.) that may impair their quality of life or psycho-behavioral status.	DLQI*^3^, SCL-90*^16^ SAD Scale*^17^ MCMQ*^18^	Social avoidance in women was higher than in men (5.69 ± 3.39 vs. 4.35 ± 3.24, t = 2.156, *p* = 0.033). Social Distress in women was higher than in men but not significatively (5.56 ± 3.83 vs. 4.63 ± 3.40, *p* = 0.179). SADS total score in women was higher than in men but not significatively (11.25 ± 6.58 vs. 8.98 ± 5.91, *p* = 0.057). SCL-90 Total Score in women was higher than in men (143.22 ± 43.66 vs. 127.02 ± 29.91, *p* = 0.019) Depression in women was higher than men (1.74 ± 0.69 vs. 1.35 ± 0.37, *p* < 0.001) Anxiety in women was higher than in men (1.67 ± 0.62 vs. 1.47 ± 0.47, *p* = 0.047) Phobia in women was higher than in men (1.39 ± 0.44 vs. 1.23 ± 0.27, *p* = 0.015) Gender differences in MCMQ and DLQI not available	Anger-hostility, Anxiety, Coping Strategies, Depression, Distress, Interpersonal sensibility, Obsessive-compulsive, Paranoid ideation, Phobic-anxiety, Psychoticism, Quality of Life, Social Avoidance, Somatization
Ongenae, K., Belgium 2005	Dermatology Department at the University Hospital of Ghent Overall *N* = 230 Vitiligo *N* = 102 Male *N* = 46 Female *N* = 56 Psoriasis *N* = 128 Female *N* = 47 Male *N* = 81	To quantify the burden of vitiligo by estimating health-related quality of life	Cross-sectional study	Patients recovered from the Dermatology Department database registered before June 2000 and secondly contacted by mail.	The Dutch version of the DLQI*^3^	Women reached a higher DLQI overall score (mean score 6.45) than men (mean 3.13) (*p* < 0.003). Women with vitiligo had a comparable overall DLQI than women with psoriasis (*P* < 0.001) Men with vitiligo scored a lower DLQI than men with psoriasis (*p* < 0.001).	Health-related quality of life.
Sampogna, F., Italy, 2008	Istituto Dermopatico dell’Immacolata, IDI-IRCCS, Rome Overall *N* = 181 Men N = 57 Female *N* = 124	To investigate the Quality of Life of patients with vitiligo. To identify categories at risk for high impairment. To analyze single items from Quality of Life instruments	Cross-sectional study	Patients ≥16 years old, recruited from November 2004 to May 2005.	Skindex-29, GHQ-12*^12^, TAS-20*^19^	Female gender associated with higher scores on Skindex-29 items #2, #6, #11, #12, #13, #15, #26, #28, and for the whole emotions and functioning subscale, *p* < 0.05.	Alexithymia, Depression, General Quality of Life, Loss of sleep, Relationship quality, Shame, Worry, Angry, Humiliation, Emotional tension.
Schmid-Ott, G., Germany, 2007	Clinic for Dermatology, Castle of Friedensburg, Leutenberg, Thüringen. Overall: *N* = 363 Male *N* = 79 Female *N* = 284 *N* = 47 current inpatients *N* = 316 former inpatients	To examine the extent of stigmatization experienced by vitiligo patients considering the visibility of the lesions. To measure how women and men suffering from vitiligo differ in their feelings of stigmatization and coping strategies and what is the modifying effect of the sense of coherence regarding the coping and stigmatization experience of this patient	Cross-sectional	Patients ≥16 years old	QES*^20^ ACS*^21^ SOC^*22^	Women were retreated more frequently and were more worried in the context of their skin disease than men. *T*-tests revealed a significant influence of gender on the QES “stigmatization retreat” (*P* = 0.03) and “stigmatization composure” (*p* < 0.001) scales. ACS revealed significantly lower results for men on the “helplessness" scale and itch-scratch circle coping strategies (*p* < 0.01).	Coping capacity, Stigmatization, Helplessness
Trapp, E., M., Austria 2015	Department of Dermatology, Medical University of Graz Patients: Overall *N* = 24 Male *N* = 8 Female *N* = 16 Healthy controls: *N* = 24	To assess the autonomic nervous tone during different standardized conditions (i) at rest, (ii) during a mental stress task and (iii) during a physical stress task in GV patients as compared to age and gender-matched controls who were not affected by a skin disease	Cross-sectional, exploratory age and gender matched 1:1 case control study. Vitiligo Patients vs. Healthy controls	Inclusion criteria: Age between 18 and 75 years, sufficient knowledge of the German language, understanding of the tasks to be performed and declared interest in taking part in the study, in the presence of hypothyroidism adequate treatment	ECG-HRV*^23^ assessment Mental stress task—d2 test of attention Physical stress task (ergo)	“Gender” variable used as control variable, no significant differences were found	Psycho-physiologic arousal during a rest in state task.

*^1^VASI, Vitiligo Area Severity Index; *^2^VitiQoL, Vitiligo Quality of Life; *^3^DLQI, Dermatology Life Quality Index; ^*4^MASI, melasma area and severity index; *^5^DPASI plus, ADMH severity scale plus; *^6^PRIME-MD PHQ, Primary Care Evaluation of Mental Disorders- Patient Health Questionnaire; *^7^PHQ-9, Patient Health Questionnaire-9; *^8^PHQ-15, Patient Health Questionnaire-15; *^9^GAD 7, General Anxiety Disorder; *^10^RRS-VR, Ruminative Response Scale; ^*11^DASS21, Depression, Anxiety, and Stress Scale; *^12^GHQ-12, General Health Questionnaire – 12; *^13^ACS, Adjustment to Chronic Skin Disorders Questionnaire; *^14^BDI, Beck Depression Inventory;*^15^MHQ, Middlesex Health Questionnaire; *^16^Symptom Checklist-90; *^17^SAD Scale, Social Avoidance and Distress Scale; *^18^MCMQ, Medical Coping Modes Questionnaire; ^19^TAS-20, 20-item Toronto Alexithymia Scale; *^20^QUES, The questionnaire on experience with skin complaints; *^21^ACS, the adjustment questionnaire to chronic skin disorders; *^22^SOC, Sense of Coherence Questionnaire; *^23^ECG-HRV, Electrocardiogram-Heart Rate Variability.

**Table 2:** Study assessment of included study using the Newcastle-Ottawa Scale adapted for cross-sectional studies.

Author	#1	#2	#3	#4	#5	#6	#7	Total
Abdullahi, U.	1	0	0	2	0	1	1	5
Al Robaee A. A.	0	0	0	2	0	1	1	4
Dabas, G.	0	0	0	2	0	1	1	4
Do Bú, E.	0	1	0	2	0	1	1	5
Ingordo, V.	0	0	0	2	1	1	0	4
Kent, G.	0	0	0	2	0	1	0	3
Krüger, C.	1	0	1	2	0	1	1	6
Ning, X.,	0	0	1	2	1	1	1	6
Ongenae, K.	0	0	0	2	1	1	1	5
Sampogna, F.	0	0	1	2	1	1	1	6
Schmid-Ott, G.	1	0	1	2	0	1	1	5
Trapp, E., M.	0	0	0	2	1	2	1	6

1. Representativeness of the case; 2. Sample size; 3. Non-Response rate; 4. Ascertainment of the screening/surveillance tool; 5. The potential confounders were investigated by subgroup analysis or multivariable analysis; 6. Assessment of the outcome; 7. Statistical test.

### Features of included findings

All collected records are cross-sectional, observational studies, except for Trapp et al. (2015) that is described as age and gender matched “exploratory case control study,” respectively. The total sample collected in this review includes 2,264 participants with vitiligo; 91 with Acquired Dermatological Macular Hyperpigmentation; 86 with Melasma; 128 with Psoriasis; and 99 were healthy controls.

In line with the purpose of this review, we focused only on data related to the people who suffered from vitiligo. Females were 1,568 (66.0%),the minimum age of the analyzed sample was attested at 16 years old, the maximum age was attested at 84 (Ingordo et al., 2014). Four studies of 12 (Kent and Al'Abadie, 1996; Schmid-Ott et al., 2007; Sampogna et al., 2008; Ingordo et al., 2014) recruited, among adult, also pediatric population. In Table 1 we described in detail the sample population for each selected study.

### Characteristics of quality of life assessment tools used in screened publications

The scientific contributions analyzed in this review reveal the wide heterogeneity of the tools used to investigate the Health-Related Quality of Life (HRQoL) and psychological symptoms among people suffering from Vitiligo. All the questionnaires used through the studies are self-administered, most of them are adapted and validated in the language of the population to which they refer.

The detailed features of these tools have been reported in Table 3.

**Table 3:** Detailed description of the assessment tools from psychosocial studies on gender differences in vitiligo included in this review.

Assessment tools	Aims	Items and subscales	Scoring methods	Used language adaptations and original versions
Adjustment to Chronic Skin Disorders (ACS)	To assess psychosocial adjustment to chronic skin disorders.	51 Items 6 subscales: 1. social anxiety/avoidance; 2. itch–scratch cycle; 3. helplessness; 4. anxious–depressive mood; 5. impact on quality of life; 6: deficit in active coping	5-Likert point scale	Krüger and Schallreuter (2015) (German version) Schmid-Ott et al. (2007) (German version) Stangier et al. (2003) (Original version)
The Dermatology Life Quality Index (DLQI)	To measure the effects of skin disease on patients’ health-related quality of life.	10 Items single scale	4-Likert point scale	Al Robaee (2007) (Arabic version) Dabas et al. (2020) (English, Hindi and Punjabi version) Ingordo et al. (2014) (Italian version) Krüger and Schallreuter (2015) (German version) Ning et al. (2022) (Chinese version) Ongenae et al. (2005) (Dutch version) Kent and Al'Abadie (1996) (English version) Finlay and Khan (1994) (Original version)
The-12 item General Health Questionnaire (GHQ-12)	To assess the risk of psychological distress and detects current non-psychotic psychiatric disorders	12 Items single scale	Two scoring methods: dichotomous (0–0–1–1), and 4-Likert point scale.	Sampogna et al. (2008) (Italian version) Goldberg et al. (1997) (Original version) Picardi et al. (2001a) (Italian adaptation)
The Questionnaire on Experience with Skin Complaints (QUES)	To identify the dimensions of stigma experienced by people with skin disease	38 Items 6 subscales: 1. worthlessness; 2. loneliness; 3. uncleanliness 4. lack of physical attractiveness or sexual desirability; 5. ways of clothing; 6. avoidance of public situations	6-Likert point scale	Schmid-Ott et al. (1998) (German version) Müller et al. (2007) (Original version)
The *Skindex-29*	To measure the effects of skin disease on patients’ health-related quality of life.	29 Items 3 subscales: 1. emotional, 2. functioning, 3. symptoms domains.	5-Likert point scale	Sampogna et al. (2008) (Italian version) Chren et al. (1996) and Nijsten et al. (2009) (Original version) Abeni et al. (2001) (Italian adaptation)
The *Vitiligo Quality of Life* (VitiQoL)	To assess quality of life of patients with diagnosis of vitiligo	15 Items single scale	7-Likert point scale	Abdullahi et al. (2021) (English version). Lilly et al. (2013) (Original version)
The *20-item Toronto Alexithymia Scale* (TAS-20)	To assess alexithymia	20 Items 3 subscales: 1. difficulty identifying feelings; 2. difficulty describing feelings to others; 3. externally oriented thinking.	5-Likert point scale	Sampogna et al. (2008) (Italian version) Taylor et al. (2003) (Original version)
The Beck Depression Inventory (BDI)	To assess the frequency of 21 symptoms and attitudes frequently experienced by depressed psychiatric patients	21 Item measuring 21 symptoms: Mood, Pessimism, Sense of Failure, Lack of Satisfaction, Guilt Feelings, Sense of Punishment, Self-dislike, Self-accusation, Suicidal Wishes, Crying, Irritability, Social Withdrawal, Indecisiveness, Distortion of Body Image, Work Inhibition, Sleep Disturbance, Fatigability, Loss of Appetite, Weight Loss, Somatic Preoccupation, and Loss of Libido	4-Likert point scale	Beck et al. (1988) and Krüger and Schallreuter (2015) (original version)
The *Big Five Questionnaire* (BFQ)	To assess the constellation of traits defined by the Five Factor Theory of Personality	132 Items 5 subscales: 1. openness, that is characterized by originality, curiosity, and ingenuity. 2. conscientiousness, that is related to orderliness, responsibility, and dependability. 3. extraversion that stands for talkativeness, assertiveness, and energy. 4. agreeableness that is characterized by cooperativeness, and trust. 5. neuroticism that refers to mood dysregulation, and is the opposite of emotional stability	5-Likert point scale	Do Bú et al. (2022) (Brazilian version) Costa and McCrae (1992) and Caprara et al. (1993) (Original version)
The *Depression, Anxiety, and Stress Scale* (DASS-21)	To assess depression, anxiety, and stress symptomatology	21 Items 3 subscales: 1. depression; 2. anxiety; 3. stress	4-point Likert scale	Do Bú et al. (2022) (Brazilian version) Henry and Crawford (2005) (Original version)
The *General Anxiety Disorder* (GAD-7)	To assess generalized anxiety disorder symptomatology	7 Items single scale	4-point Likert scale	Dabas et al. (2020) (English, Hindie and Punjabi version). Spitzer et al. (2006) (original version)
The *Medical Coping Modes Questionnaire* (MCMQ)	To assess three cognitive–behavioral, illness-related coping strategies	20 Items 3 subscales: 1. confrontation; 2. avoidance; 3. acceptance–resignation	4-point Likert scale	Ning et al. (2022) (The formal Chinese version combined with the original Chinese translation version) Feifel et al. (1987) (Original version)
The *Primary Care Evaluation of Mental Disorders- Patient Health Questionnaire* (PRIM- MD PHQ)	To assess psychiatric disorders according to DSM-IV	PRIMEMD 26 Items 7 domains: 1. somatoform disorders; 2. major depressive syndrome; 3. other depressive syndrome; 4. panic syndrome; 5. bulimia nervosa; 6. binge eating disorder; 7. alcohol abuse PHQ-9 9 Items: depressive symptoms PHQ-15 15 Items: somatic symptoms	The item rating is mixed (some items are rated on a 3- and 4-point Likert scale, other are dichotomous: Yes/No)	Dabas et al. (2020) (English version) of PRIME-MED PHQ with PHQ-9 and PHQ-15 modules. PRIMEMD Spitzer et al. (2006) (Original version) PHQ-9 Manea et al. (2015) (Original version) PHQ-15 Kroenke et al. (2002) (Original version)
The *Ruminative Response Scale* (RRS-VR)	To measure two factors (brooding and reflection) of depressive rumination according to the theoretical framework of the Response Theory Style	10 Items 2 subscales: 1. brooding; 2. reflection	4-point Likert scale	do Bú et al. (2022) (Portuguese version) Zanon et al. (2018) (Portuguese adaptation) Nolen-Hoeksema (2000) (Original version)
The *Sense of Coherence Questionnaire* (SOC)	To assess the ability to perceive the relationship between actions and consequences in everyday life and the ability to perceive the meaning of the world in a clear and structured way	13 Items 3 subscales: 1. comprehensibility; 2. manageability, 3. meaningfulness	7-point Likert scale	Schmid-Ott et al. (2007) (German version) Antonovsky (1993) (Original version)
The *Social Avoidance and Distress Scale* (SAD scale)	To measure aspects of social anxiety including distress, discomfort, fear and avoidance	28 items single scale	Dichotomous (i.e., true/false)	Ning et al. (2022) (Chinese version) Watson and Friend (1969) (Original version)
The *Symptom Checklist-90* (SCL-90)	To evaluate a broad range of psychological problems and symptoms of psychopathology	90 Items 9 scales: 1. somatization; 2. obsessive-compulsive behavior; 3. interpersonal sensitivity; 4. depression; 5. anxiety; 6. hostility; 6. phobic anxiety; 7. paranoid ideation; 8. psychoticism. 10. a variety of symptoms related to sleep and appetite disturbances.	5-point Likert scale	Ning et al. (2022) (Chinese version) Derogatis and Cleary (1977) (Original version)
*Electrocardiogram Heart Rate Variability* (ECG-HRV)	To evaluate stress levels of cardiovascular system	It is performed on inter-beat intervals obtained from the R waves in electrocardiogram.	–	Saykrs (1973), Perini and Veicsteinas (2003), and Trapp et al. (2015) (Original version)

### Differences among genders detected in skin disease quality of life measurements

A statistically significant difference in HRQoL, was reported in only two of the seven eligible studies that used DLQI (Kent and Al'Abadie, 1996; Ongenae et al., 2005; Al Robaee, 2007; Ingordo et al., 2014; Krüger and Schallreuter, 2015; Dabas et al., 2020; Ning et al., 2022). Ingordo et al. (2014) detected a significantly higher frequency of DLQI>5 in women than men (Ingordo et al., 2014). Also Ongenae et al. (2005) reported significant higher DLQI mean score in women than men (6.45 vs. 3.13, *p* < 0.001) (Ongenae et al., 2005). In the other five studies there were no significant differences in DLQI mean scores among genders.

The VitiQol, the disease-specific tool for vitiligo, used in a single study of those eligible (Abdullahi et al., 2021), showed that the HRQoL was significantly more impaired in women than men (*p* = 0.037), with the stigma component as the major contributor to the high mean VitiQoL (p < 0.001).

The other HRQoL tool used in another single study of the eligible (Sampogna et al., 2008) was the Skindex-29. In this study, Sampogna et al. (2008) found a significant difference in Skindex-29 scores, with worse outcomes for women in items #2, #6, #11, #12, #13, #15, #26, #28, and for the whole emotions and functioning subscale (*p* < 0.05), but not for the symptom’s subscale. This result is in accordance with previous research on gender differences in chronic autoimmune dermatology patients (Samela et al., 2023).

### Differences among genders detected in psychological symptomatology assessment tools

The studies identified in this systematic review used a huge range of psychologic tools, and explore several psychologic features, both dispositional and situational. For an extensive description of constructs assessed in each study (see Table 1).

## Discussion

The aim of our systematic review, conducted according to the PRISMA guidelines, was to assess the differences among genders in HRQoL and in psychologic symptoms among persons with vitiligo. The qualitative assessment of the 12 eligible studies (Kent and Al'Abadie, 1996; Ongenae et al., 2005; Al Robaee, 2007; Schmid-Ott et al., 2007; Sampogna et al., 2008; Ingordo et al., 2014; Krüger and Schallreuter, 2015; Trapp et al., 2015; Dabas et al., 2020; Abdullahi et al., 2021; Do Bú et al., 2022; Ning et al., 2022) revealed a wide heterogeneity in term of methodology, as well as in terms of quality among studies. The design of all the studies included in the review was cross-sectional. Also, the pediatric population was included in several reports. For these reasons we chose to discuss our results by symptom or characteristic, rather than by assessment tool.

### Personality traits

Personality traits in patients with vitiligo were assessed in only one study (Do Bú et al., 2022). Their results highlighted the presence of significant differences between genders, in fact women tended to present higher level of neuroticism than men, as also occurs in the general population (Lynn and Martin, 1997). However, high levels of neuroticism could interfere with the patients’ psychological adaptation to vitiligo (Bonotis et al., 2016), and could increase the risk of a worse adaptation to the disease for women than men.

### Alexithymia

Although the psychoanalytic tradition has identified several types of alexithymia (Sifneos, 1973; Freyberger, 1977; Honkalampi et al., 2000), and Honkalampi et al. (2000) have identified changes in the levels of alexithymia over time, Sifneos (1973) and other recent authors have defined alexithymia as a personality trait (Kumari et al., 2023). Beyond the unresolved theoretical discussion, in our results only one study, by Sampogna et al. (2008), assessed alexithymia in persons with vitiligo, finding a higher prevalence of this trait in their patients (i.e., 24%), compared to the general populations (approximately 10%). No differences among genders were detected. This result is in contrast with what is asserted in the literature for the general population, in which usually men score higher than women on measures of alexithymia (Levant et al., 2009).

### Coping strategies

Two studies assessed coping strategies in persons with vitiligo (Schmid-Ott et al., 2007; Krüger and Schallreuter, 2015) using the ACS. Krüger and Schallreuter (2015) found that female patients tend to have worse coping strategies than men, particularly in the ACS subscale that assesses emotional and physical symptomatology of general disturbance in coping with the emotional distress because of the disease. Conversely, Schmid-Ott et al. (2007) found worse coping ability in men than women, especially in Itch–Scratch Cycle (which reflects the inability to cope with itching, and feelings of helplessness concerning the cycle of itching and scratching) and Helplessness (that concerns the experience of loss of hope about the recurrence of the skin condition).

### Stigmatization

The experience of stigmatization, evaluated through the VitiQol (Abdullahi et al., 2021) and the QUES (Schmid-Ott et al., 2007). Schmid-Ott et al. (2007), showed that women tend to experience a lack of self-perceived physical attractiveness or sexual desirability because of the skin disease more frequently than men. They were also more worried than men about their skin disease. It is possible to hypothesize that women struggle more about their skin problems because appearance norms for women are more rigid, homogeneous, and pervasive than those for men (Buote et al., 2011). Everyday exposure to norms and social pressure may exacerbate negative emotions, maladaptive cognitions, with worse negative mental health outcomes in women (Łakuta et al., 2017).

### Rumination

Only one study (Do Bú et al., 2022) highlighted the presence of high levels of rumination among persons who suffered from vitiligo, in fact both women and men presented high levels of this manifestation. However, for women, stress and reflection (i.e., a rumination component) seem to be important mechanisms in predicting anxiety and depression symptoms, whereas brooding (i.e., the other rumination component) is associated with the same symptomatology in men. According to these results, it is possible to hypothesize that women tend to engage cognitive problem-solving strategies to struggle with depressive and anxiety symptoms, whereas men seem to adopt strategies more related to a passive comparison of their current situation with some unachieved standard, in dealing with depression and anxiety (Verstraeten et al., 2010).

### Depression

Depression has been evaluated through different tools in five of the eligible studies. Only Dabas et al. (2020) did not describe differences among genders in this clinical variable. Conversely, Sampogna et al. (2008), Krüger and Schallreuter (2015), Do Bú et al. (2022), and Ning et al. (2022), and consistently highlighted a significant higher score in depression symptomatology in women than in men. A comprehensive meta-analysis by Wang et al. (2018) confirmed that female patients with vitiligo were more likely to show depression when compared with male patients. It is well known that depression accounts for a substantial part of the psychosocial burden of chronic disorders (Gold et al., 2020). However, concerning the specific case of skin disorders, feelings of shame could increase social avoidance behaviors and withdrawal, which, in turn, might result in reduced perceived social support, and increasing hopelessness. Helplessness is, in fact, also detected by Schmid-Ott et al. (2007) and is significantly higher in women than men.

### Anxiety

The general prevalence of anxiety among vitiligo patients worldwide (i.e., 35.8%) was comparable to other severe skin disorders (Kussainova et al., 2020), and is significantly higher in females than in males (Kussainova et al., 2020; Liu et al., 2021). This difference has been confirmed in Do Bú et al. (2022) and Ning et al. (2022), but not in Dabas et al. (2020). It is possible to deduce that patients with vitiligo experienced a substantial burden because of anxiety (Liu et al., 2021). Several hypotheses may explain the increased prevalence of anxiety disorders among individuals with vitiligo; for example, female patients with skin depigmentation suffer from more discrimination in daily life (Chaturvedi et al., 2005). In partial accordance with our data, and according to the literature, females could be at higher risk of developing social anxiety symptoms, due to greater appearance awareness, feeling of looking unattractive, and lower self-confidence (Sawant et al., 2019). These negative effects, according to the Cognitive Behavioral model of social anxiety, could result in avoidance behaviors (e.g., avoid meeting new people, being sexually inhibited), that bolster social anxiety symptomatology (Heimberg et al., 2010).

### Stress

A relevant corpus in the literature points the attention on psychological stressors that may play a role in vitiligo and, in general, in skin disorders (Papadopoulos et al., 1998; Silverberg and Silverberg, 2015), even if this cause-effect mechanism is controversial (Picardi and Abeni, 2001). Stressful life events have been considered as antecedent factors to the onset of vitiligo (Silverberg and Silverberg, 2015). Moreover, from a bio-psychological point of view, chronic stress could have a role in the pathogenesis of vitiligo (Miniati et al., 2012; Cupertino et al., 2017). In neither of selected study biological stress-related parameters of hormonal imbalances were detected, but it has been observed the presence of high levels of distress in vitiligo female patients compared to the test norms (Do Bú et al., 2022; Ning et al., 2022). Only one study reported significantly higher stress levels in women than men (Do Bú et al., 2022).

To the best of our knowledge, and based on what emerged from our systematic review, women with vitiligo have a higher risk to experience low quality of life, and worse mental health in a wide range of psychopathology symptoms than men. Unfortunately, there are few explanatory models proposed in the literature to rationalize the greater prevalence of psychopathological symptoms in women compared to men in vitiligo. This could be explained by the cross-sectional nature of the considered studies, and because of the used assessment methods, which mainly investigate the presence of these symptoms only at the moment of assessment, not exploring the factors that may affect the emergence of these issues. It will be important to investigate in further researches the specific influence of, for example, the disease duration, comorbidity, socio-demographic features, personality traits, components of attachment behavior, and other known risk factors for psychopathology in this patient population to better explore these phenomena.

### Study limitations and conclusion

It is important to note that some issues limit the generalizability of our findings. A single search string was used in the databases used for our systematic review. No gray literature databases have been considered. We tried to minimize publication bias by rigorously following internationally accepted criteria of study selection for systematic reviews. No data referring to non-binary or gender-fluid patients have been identified in the included studies. Very few studies report the word “gender” or “gender differences” in the aims or in the keywords. Most differences in the scores between the genders reported in this paper were abstracted from one single eligible article.

Our study differed from previous studies and added to previous findings by investigating directly the most used tools to study QoL and psychopathological symptoms in people who suffer from vitiligo, with a specific focus on gender differences. Such differences seem to exist, in fact, with most reports showing a larger impact of vitiligo on the psychological well-being among women than in men. However, the vast methodological heterogeneity of the reports, e.g., in terms of study population, sample size, investigated construct, patient-reported outcomes tools used—does not allow to perform a meta-analysis or to compute summary measures such as effect sizes. Not only that, but such heterogeneity is so extreme that each investigated construct is based on an insufficient number of studies (i.e., often in a single study) to make evidence-based conclusions about the validity and generalizability of these findings.

Given the relevant impact of vitiligo on the psychosocial wellbeing of patients, it would be advisable to identify a standardized core of patient-reported outcomes so that more meaningful comparisons may be made across different studies.

## Data availability statement

The raw data supporting the conclusions of this article will be made available by the authors, without undue reservation.

## Author contributions

WM, TS, and DA: conceptualization. TS and SA: data curation, methodology, formal analysis, writing, and revise the original draft. WM and DA: project administration and supervision. All authors contributed to the article and approved the submitted version.
